# Analysis of risk factors and changes in myocardial biomarker levels in 122 cases of early neonatal anemia

**DOI:** 10.55730/1300-0144.5788

**Published:** 2023-12-18

**Authors:** Zhenhua SUI

**Affiliations:** Department of Clinical Laboratory, Tianjin Central Hospital of Gynecology Obstetrics, Tianjin, China

**Keywords:** Early neonatal anemia, risk factors, myocardial markers

## Abstract

**Background/aim:**

Anemia in the first week after birth, which could affect growth, development, and organ function, should be an important warning sign to clinicians. The aim of this study was to assess the related risk factors of early neonatal anemia and to analyze the effect of anemia on the expression levels of myocardial markers in newborns.

**Materials and methods:**

Clinical data from 122 confirmed cases of anemic newborns and 108 nonanemic newborns were collected to analyze the independent risk factors for early anemia using logistic regression analyses. Blood samples were collected from both groups for the detection of myocardial markers, including the protein marker cardiac troponin T (cTnT), as well as enzyme markers creatine kinase isoenzyme MB (CK-MB) and lactate dehydrogenase (LDH).

**Results:**

Multivariate logistic regression analysis revealed that preterm birth (OR: 3.589 [1.119–11.506], p < 0.05), multiple pregnancy (OR: 4.117 [1.021–16.611], p < 0.05), and abnormal placenta (OR: 4.712 [1.077–20.625], p < 0.05) were independent risk factors for early neonatal anemia. The levels of myocardial markers, including cTnT (303.1 ± 244.7 vs. 44.2 ± 55.41 ng/L), CK-MB (6.803 ± 8.971 vs. 2.5326 ± 2.927 μkat/L), and LDH (32.42 ± 35.26 vs. 19.73 ± 17.13 μkat/L), were significantly higher in the anemic group than in the nonanemic group.

**Conclusion:**

Multiple pregnancy, preterm birth, and abnormal placenta were identified as risk factors for early neonatal anemia. The occurrence of early neonatal anemia was associated with increased levels of myocardial markers.

## 1. Introduction

According to the World Health Organization’s report, approximately a quarter of the world’s population suffers from anemia, with almost half of preschool-age children affected [1]. Anemia in newborns presents a more complex issue due to the special physiological period. Currently, there is no globally standardized criterion for diagnosing neonatal anemia. In some countries, anemia severity is assessed based on hemoglobin (Hb) or hematocrit (HCT) levels lower than two standard deviations from normal age-matched controls after birth [2]. In China, the diagnosis standards for anemia involve Hb ≤130 g/L and HCT ≤ 0.43 in venous blood within 2 weeks after birth, as well as Hb ≤145 g/L and red blood cell count <4.6 × 10^12^/L in peripheral blood. Anemia occurring within 7 days after birth is considered early anemia, while that occurring more than 7 days later is classified as late anemia [3,4]. Neonatal anemia can lead to significant tissue hypoxia and organ dysfunction [5], and may even result in delayed neural development and brain damage [6].

The long-term consequences of anemia can lead to developmental delay, cognitive impairment, poor social-emotional development, as well as increased susceptibility to infection due to chronic hypoxia [7]. Anemia has multifactorial causes; studies have indicated that malnutrition is the primary cause in children under 5 years old [8], while another study identified infection as another major cause of childhood anemia [9]. However, research on risk factors for early neonatal anemia remains limited. Furthermore, beyond the various tissue and organ damages mentioned above induced by anemia, the relationship between early neonatal anemia and myocardial biomarker levels is still unclear. This study collected cases of early anemia (peripheral blood Hb ≤ 145 g/L and HCT ≤ 0.43 within the first week after birth) according to domestic standards combined with clinical practice, exploring the risk factors for early neonatal anemia and investigating the relationship between early neonatal anemia and myocardial biomarkers.

## 2. Materials and methods

### 2.1. Study subjects

This study included 122 newborns diagnosed with neonatal anemia who were hospitalized in the neonatology department of Tianjin Central Hospital of Gynecology and Obstetrics from January 2019 to December 2022. The inclusion criteria were peripheral blood Hb ≤145 g/L and HCT ≤0.43 at any time during the first week after birth. A control group, comprising 108 nonanemic newborns admitted during the same period, was selected.

### 2.2. Definition of risk factors

Advanced maternal age was defined as maternal age ≥35 years old. Abnormal placenta included placental abruption, placenta accreta, and placenta previa. Pregnancy anemia was defined as Hb <110 g/L during pregnancy. Multiple pregnancy referred to pregnancy with two or more fetuses in a single pregnancy cycle. Pregnancy complications included gestational diabetes mellitus, gestational hypertension, and hypothyroidism during pregnancy. Preterm birth was defined as a gestational age <37 weeks, while low birth weight referred to a newborn weighing less than 2500 g at birth.

### 2.3. Laboratory index detection

Out of the 122 cases diagnosed with neonatal anemia in this study, we excluded 69 cases diagnosed with perinatal asphyxia, respiratory distress syndrome (RDS), arrhythmia, or severe infection. This exclusion was made considering that these diseases themselves could affect cardiac biomarker activity levels. Consequently, only 53 cases were enrolled for further analysis. Plasma samples were collected through venipuncture within 24 h after birth using tubes containing separation gel-coagulant, following the instructions provided by Roche Diagnostics GmbH (Mannheim, Germany). The cTnT level was measured using a Roche Elecsys 411 analyzer and the Roche troponin T test kit (Lot code: 09315349190). The Elecsycs Troponin T hs STAT kit used in this study could be traced to Troponin T (CARDIACT) enzyme-linked immunosorbent assay (ELISA). The CK-MB and LDH levels were measured using a Roche Cobas c8002 analyzer, along with supporting reagents, calibration products, and quality control products. Cardiac biomarker levels in 48 newborns from the control group were also detected during the same period.

### 2.4. Statistical analysis

Statistical analysis was performed using GraphPad Prism 7.0 and SPSS (v22, IBM, Chicago, IL, USA, 2013). Group comparisons were carried out using chi-squared tests and t-tests. Multivariate logistic regression analysis was used to analyze risk factors. Quantitative variables were presented as mean ± standard deviation and analyzed using an independent t-test. A significance level of p < 0.05 was considered statistically significant.

## 3. Results

### 3.1. General information of the two groups of newborns

In the study, 122 newborns with early anemia and 108 nonanemic newborns were included. The general conditions of the two groups are presented in Table. Neonatal weight in the control group was significantly higher than that in the anemia group (p < 0.05). The Hb (86.17 ± 5.41 g/L vs. 197.70 ± 8.41 g/L, p < 0.05) and HCT (0.278 ± 0.02 vs. 0.579 ± 0.02, p < 0.05) levels in the newborns of the anemia group were significantly lower than those in the control group. There was no statistically significant difference between the two groups in terms of sex and 1 min Apgar score comparison (p > 0.05).

### 3.2. Analysis of risk factors of early neonatal anemia

**Figure 1:**
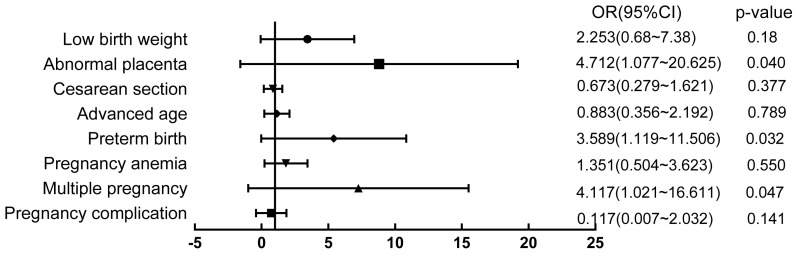
Analysis of risk factors for neonatal anemia. p < 0.05 was considered as an independent risk factor for early neonatal anemia.

Logistic regression analysis was used to analyze the perinatal risk factors of early neonatal anemia. As depicted in Figure 1, preterm birth (OR: 3.589 [1.119–11.506], p < 0.05), multiple pregnancy (OR: 4.117 [1.021–16.611], p < 0.05), and abnormal placenta (OR: 4.712 [1.077–20.625], p < 0.05) were independent risk factors for early neonatal anemia. There were no differences in maternal age, complications, mode of delivery, pregnancy anemia, and low birth weight between early anemia newborns and nonanemic newborns (p > 0.05).

### 3.3. Analysis of levels of cardiac markers in two groups of newborns

**Figure 2:**
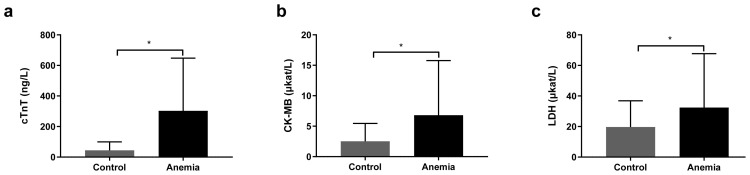
Analysis of levels of cardiac markers in anemic and nonanemic newborns. cTnT, serum cardiac troponin T (newborns reference range: <100 ng/L); CK-MB, creatine kinase isoenzyme MB (newborns reference range: 0.84~5.18 μkat/L); LDH, lactate dehydrogenase (newborns reference range: 7.16~17.45 μkat/L). * p < 0.05.

To investigate whether anemia affected the level of cardiac markers, the cTnT, CK-MB, and LDH levels were detected in 53 eligible anemia newborns and 48 nonanemia newborns. Comparative findings of laboratory examinations between the two groups were displayed in Figure 2a. In anemia newborns, the cTnT level (303.1 ± 244.7 ng/L) was higher than that in the control group (44.2 ± 55.41 ng/L). Meanwhile, anemia newborns showed higher levels of CK-MB (Figure 2b) and LDH (Figure 2c) compared with the control group.

## 4. Discussion

The neonatal period is the most dynamic stage, marked by profound changes and adjustments as the newborn transitions from a dependent intrauterine life to an independent one. Anemia that occurs within the first week after birth serves as a crucial warning signal, influencing organ function and growth in newborns. Therefore, early anemia in newborns should be given adequate attention.

This study suggests that preterm birth is one of the risk factors for early neonatal anemia. A previous cross-sectional study supports this finding, indicating that the incidence of anemia in premature infants is significantly higher than that in full-term infants (38.5% vs. 10.2%) [10]. The immature hematopoietic system of premature infants, with fewer red blood cells and shorter lifespans, increases the risk of anemia [11]. The underdeveloped immune system of premature infants also makes them more susceptible to infections, which can cause anemia. In addition to preterm birth, this study found that multiple pregnancy was also an important risk factor for early neonatal anemia. Moreover, twin-twin transfusion syndrome (TTTS) was identified as a significant cause of newborn anemia during multiple pregnancy. TTTS results from imbalanced oxygen and nutrient supply between twins through placental blood vessels’ fusion sites, leading to fetal anemia [12]. With the development of assisted reproductive technology, the incidence rate of multiple pregnancy has been continuously increasing. Therefore, clinical attention should be directed towards factors affecting newborn health, such as TTTS. However, another study suggested the presence of hypoxia in the donor placenta during TTTS, yet vascular volume loss and subsequent renin-angiotensin system (RAS) activation resulted in a concentrated blood state, making it unlikely for anemic conditions to occur [13]. This finding necessitates further analysis and research. This study also found that abnormalities such as placenta previa, placental abruption, or malformations like placenta implantation may cause anemia. This contradicts previous views suggesting that placental abruption does not cause neonatal anemia [14] and that only maternal blood is lost during placental abruption, not fetal blood [15]. However, Jang et al. supported the view of this study, suggesting that placenta previa is an independent risk factor for neonatal anemia [16]. Abnormalities in the placenta may disrupt blood circulation between the placenta and the uterus, leading to anemia by affecting oxygen and blood supply to the fetus. However, all of these hypotheses require further investigation.

This study also revealed that early neonatal anemia could increase the expression levels of myocardial biomarkers. Clinical observations have demonstrated that neonatal asphyxia and respiratory distress can also elevate myocardial marker levels due to myocardial cell damage caused by myocardial ischemia or hypoxia. Severe infections in newborns, by infecting myocardial cells, can lead to increased myocardial biomarkers. Consequently, this study excluded the aforementioned cases and focused solely on how anemia affected myocardial markers.CK-MB and cTnT are crucial indicators for detecting cardiac injury. CK-MB primarily exists in the cytoplasm of cardiac cells, reflecting the integrity of cardiac cells [17]. However, its specificity is limited, and it is not sensitive to minor injuries to the heart muscle. On the other hand, cTnT exhibits high specificity and sensitivity for the heart muscle, making it a superior marker for diagnosing cardiac injury [18,19]. cTnT is considered a gold standard clinical diagnostic biomarker for acute myocardial infarction [20], and high-sensitivity cTnT is identified as a risk factor for major adverse cardiovascular events and all-cause mortality [21]. The findings of this study further confirmed that both CK-MB and cTnT levels were significantly higher in neonates with anemia compared to those without anemia. Another myocardial biomarker, LDH, is widely present in various tissues. Its specificity for diagnosing cardiac injury is low, and elevated above normal levels were observed in both groups studied here.

The increase in myocardial marker levels caused by anemia may be attributed to the reduced oxygen-carrying capacity of blood during anemic conditions. When blood delivers less oxygen, it can result in hypoxic necrosis of cardiomyocytes, releasing these markers into circulation. Additionally, a reduction in ATP production may affect membrane stability under insufficient oxygen supply, leading to the release of these markers into circulation as well [22].

This study has certain limitations. Firstly, it relied on a single-center sample with a limited number of cases. Further validation through multicenter large-sample studies would be necessary before generalizing the findings. Secondly, the study only examined changes in myocardial markers, and whether alterations in parameters such as ejection fraction (EF) or stroke volume (SV) accompany potential heart damage has not been verified. In conclusion, this study identified multiple pregnancy, preterm birth, and abnormal placenta as risk factors for early neonatal anemia. Early neonatal anemia was associated with increased levels of myocardial markers.

## Figures and Tables

**Table:** Comparison of general information between two groups of newborns (*p < 0.05).

Variables	Control group (n = 108)	Anemia group (n = 122)	χ^2^/*t*	p-value
Hb (g/L)	197.70 ± 8.41	86.17 ± 5.41	10.88	0.0001*
HCT	0.579 ± 0.02	0.278 ± 0.02	10.01	0.0001*
Sex (male/female)	59/49	71/51	0.297	0.586
Weight (g)	3058 ± 670	1976 ± 1009	7.63	0.001*
1 min Apgar score	9.05 ± 0.17	8.44 ± 0.26	1.996	0.0536

Hb: hemoglobin; HCT: hematocrit.
